# Digital Dependency: How Parenting and Social Intelligence Shape Internet Addiction

**DOI:** 10.1155/2023/7852467

**Published:** 2023-09-19

**Authors:** Lawrence E. Ugwu, Erhabor S. Idemudia, Maria-Chidi C. Onyedibe, Adaobi Eze, Ntasiobi C. N. Igu, Pamela Ogbozor, Francis Chuwkuemeka Chinawa

**Affiliations:** ^1^North-West University, Mafikeng, South Africa; ^2^Department of Psychology, University of Nigeria Nsukka, Enugu, Nigeria; ^3^Department of Psychology, Enugu State University of Science and Technology, Enugu, Nigeria; ^4^Alex Ekwueme Federal University Ndufu Alike Ikwo, Abakaliki, Ebonyi, Nigeria; ^5^Department of Psychology, Godfrey Okoye University, Enugu, Nigeria

## Abstract

The global pandemic forced young adults and their parents to be together. This situation has equally exposed the weaknesses in the child-parent relationship. This study aimed to investigate the role of social intelligence in the relationship between parenting style and Internet addiction during the global COVID-19 pandemic. Seven hundred and seventy-four were sampled from a public university in southeast Nigeria. They comprised 373 females and 401 males aged 17–28 years, with a mean age of 21.61. The students responded to validated measures of parenting style inventory-II, the Tromsø social intelligence scale, and Young's Internet addiction test. The moderated multiple regression analysis results indicated that permissive parenting and social intelligence significantly predicted Internet addiction. Social intelligence moderated the relationship between authoritarian parenting style and Internet addiction. The moderation was that Internet addiction is significantly higher for individuals with low social intelligence and authoritarian parenting style than individuals with low social intelligence and higher authoritarian parenting style. Some implications of the findings include engaging parenting styles to encourage more physical interactions and enabling an environment for growth. Also, adopting techniques to increase social intelligence will help students adjust to any parenting style that may influence their psychological well-being.

## 1. Introduction

The emergence of Internet addiction (IA), a behavioural addiction marked by compulsive Internet use and significant functional impairments, is an escalating concern in both developed and developing countries, particularly among adolescents and young adults [1–3].

Many find themselves leaning heavily on online platforms for various tasks in today's digital age, creating a particular digital dependency. However, for some, this dependency escalates, leading to what is recognised as Internet addiction (IA): a condition where Internet use becomes compulsive and starts affecting daily functionalities [4].

The intricate role of social intelligence (SI) in this context must be considered. SI, representing an individual's ability to navigate and understand social scenarios adeptly, has profound implications for IA. Those with heightened SI may find their social needs adequately met within real-world settings, thus reducing their reliance on the digital realm for interpersonal interactions [5]. Conversely, individuals with diminished SI might use the Internet to compensate for real-world social challenges [6]. Given that parenting styles can mould the development of SI in offspring, dissecting the triadic relationship between PS, SI, and IA becomes crucial.

It is worth noting that the DSM-5 does not currently recognise IA as an official mental disorder. However, the manual does include a section proposing Internet gaming disorder for further research, reflecting growing awareness about problematic Internet use and its potential mental health impacts [7, 8].

The spread of the novel coronavirus (COVID-19) disease has further escalated this issue due to enforced stay-at-home measures, leading to increased online activities [9]. This has been especially prevalent among college students now spending more time in home environments with their parents or guardians [10, 11].

Research has shown that aspects of family life, particularly parenting style (PS), can significantly impact adolescents' psychosocial development and behavioural patterns [12, 13]. For example, problematic family dynamics and authoritarian PS have been associated with more IA among adolescents [14–16]. PS is generally categorised into three types: authoritative, authoritarian, and permissive, each demonstrating different demands and emotional responsiveness [17]. For instance, an authoritarian parent might respond to a child's query with ‘*Because I said so*,' emphasising strict rules and obedience. On the other hand, an authoritative parent might engage in a discussion, setting boundaries while being responsive. In contrast, a permissive parent could avoid setting strict rules, acting more like a friend than an authority figure.

Simultaneously, social intelligence (SI), an individual's capacity to effectively navigate social interactions [18], has been theorised to influence IA potentially. Although direct studies linking SI and IA are lacking, related constructs such as emotional intelligence (EI) have been inversely associated with IA, suggesting that individuals with lower EI may be more prone to higher levels of IA [19]. In addition, researchers [20] have found that individuals with poor social skills or who experience a high level of interpersonal anxiety may be susceptible to cyberspace relationships [21]. This suggests that individuals with low social intelligence could be predisposed to greater IA levels than people with higher SI.

While the relationship between PS and IA has been examined in previous studies, there is a gap in the literature regarding how PS might interact with SI to influence IA. Furthermore, the role of SI as a potential moderator in the relationship between PS and IA needs further exploration. In contrast to the significant role of parent-child relationships in shaping interpersonal relationships, it is hypothesised that SI may be further cultivated among peers rather than in parent-child interactions [22].

Building upon the general strain theory posits that stress may lead to problem behaviours [23]. This theory suggests that various strains or stress experienced by adolescents might cause negative emotions, which subsequently may lead to problem behaviours. Researchers have shown that having unresponsive and controlling parents can be very stressful [24], and stress is one of the critical antecedents of IA for adolescents [25]. Internet use to relieve stress, anxiety, and depression is a strong reinforcer for adolescents. This study investigates whether SI moderates the relationship between PS and IA, proposing that controlling parents and low SI could contribute to higher levels of IA among adolescents.

### 1.1. Present Study

This research aims to bridge the gap in the literature, potentially providing valuable insights into the interplay between PS, SI, and IA. The study aims to delineate PS and SI's direct and interactive roles in IA among undergraduate students. In doing so, this research may offer crucial contributions to understanding IA and the factors that influence it, thereby providing potential avenues for its prevention and management.

## 2. Materials and Methods

### 2.1. Participants

Seven hundred and seventy-four undergraduate students (373 females and 401 males) conveniently selected from eight faculties at the University of Nigeria Nsukka participated in this study. Their ages ranged from 17 to 28 years, with a mean age of 21.61 years (SD = 3.37). The study also examined who the participants lived with before and during the COVID-19 pandemic, along with its associated restrictions and lockdown. Among the participants, 611 (78.94%) reported living with their parents, while 163 (21.06%) lived with their guardians.

### 2.2. Measures

#### 2.2.1. Parenting Style Inventory-II

PSI-II [26] is a 15-item scale that measures three forms of PS as reported by the students authoritative, authoritarian, and permissive PSs. Examples of items in the scale include the following: “My parents hardly ever praise me for doing well” and “My parents believe I have a right to my point of view.” It is designed in a 5-point Likert-type response format, ranging from 1 = strongly disagree to 5 = strongly agree. Items 1, 2, 6, 9, 12, and 15 are reverse-scored.

#### 2.2.2. The Tromsø Social Intelligence Scale

This measure is a 21-item self-report measure that assesses three areas of SI (English version) [27]. Each subscale comprises 7 items conducted as a 7-point Likert-type scale ranging from 1 = describes me extremely poor to 7 = describes me extremely well.

#### 2.2.3. Internet Addiction Test (IAT)

The IAT is a 20-item self-report scale that was rated on a Likert scale ranging from 1 (“not at all”) to 5 (“always”) and measures the degree of problematic Internet user behaviour [28].

### 2.3. Validity of the Instruments

For PSI-II, confirmatory factor analysis was conducted with IBM AMOS 21, constraining the instruments into three dimensions stated in the original instrument. The indices recommended by Schreiber et al. [29] to indicate an adequate model fit were goodness-of-fit (GFI), comparative fit index (CFI), root mean square error of approximation (RMSEA), and they yielded acceptable scores (GFI = 0.95, CFI = 0.97, TLI = 0.92, and RMSEA = 0.056).

For the Tromsø social intelligence scale, confirmatory factor analysis was conducted with IBM AMOS 21, constraining the instruments into three dimensions stated in the original instrument. The indices recommended by Schreiber et al. [29] to indicate an adequate model fit were goodness-of-fit (GFI), comparative fit index (CFI), and root mean square error of approximation (RMSEA), and they yielded acceptable scores (GFI = 0.97, CFI = 0.99, TLI = 0.95, and RMSEA = 0.044).

For the IAT, a confirmatory factor analysis was conducted with IBM AMOS 21, constraining the instruments into six dimensions stated by the authors. The indices recommended by Schreiber et al. [29] to indicate an adequate model fit were goodness-of-fit (GFI), comparative fit index (CFI), and root mean square error of approximation (RMSEA), and they yielded acceptable scores (GFI = 0.93, CFI = 0.95, TLI = 0.93, and RMSEA = 0.067).

### 2.4. Data Analysis

The data were analysed using the Statistical Package for Social Sciences (SPSS) version 20. Confirmatory factor analyses for the instruments were conducted using IBM AMOS 21. The means, standard deviations, and correlations among the study variables were computed first. Then, hierarchical multiple regression analysis was used to test the study's hypotheses.

## 3. Results

The results in Table 1 indicate that none of the demographic variables used as control variables were significantly related to IA. For the main predictors, the results also showed that permissive PS (*r* = −0.32, *p*  <  0.01) and social intelligence (*r* = −0.42, *p*  <  0.001) had a significant negative relationship with Internet addiction, whereas authoritative PS (*r* = −0.12*, p* = 0.21) and authoritarian PS (*r* = −0.06, *p* = 0.49) are not significantly related to IA.

The results of the moderated multiple regression in Table 2 indicate that the demographic variables entered in step 1 of the equation collectively accounted for 0.3% variance in Internet addiction (∆*R*^2^ = 0.003, *p* = 0.51). They all fail to predict IA significantly.

The entry of PS in step 2 of the equation contributed to a significant 4.0% in IA (Adjusted *R*^2^ = 0.04, *p*  <  0.01), with only permissive PS (*β* = −0.24, *p* = 0.01). However, authoritative PS (*β* = −0.04, *p* = 0.89) and authoritarian PS (*β* = −0.03, *p* = 0.71) did not contribute significantly. When SI was added to the third model, it contributed a significant 7.0% variance in IA, making a significant contribution (*β* = −0.32, *p*  <  0.001) to the prediction of IA. The inclusion of the interaction terms between authoritative PS and SI, permissive PS and IA, and authoritarian PS and IA in step 4 of the equation collectively accounted for a significant 9.0% variance in IA (∆*R*^2^ = 0.09, *p*  <  0.01). The interaction between authoritarian PS and SI was significant (*β* = 0.25, *p*  <  0.001). In contrast, the interaction between authoritative PS and SI (*β* = 0.06, *p* = 0.42) and permissive PS and SI (*β* = 0.08, *p* = 0.23) was not significant.

### 3.1. Parenting Style and Internet Addiction

**Figure 1:**
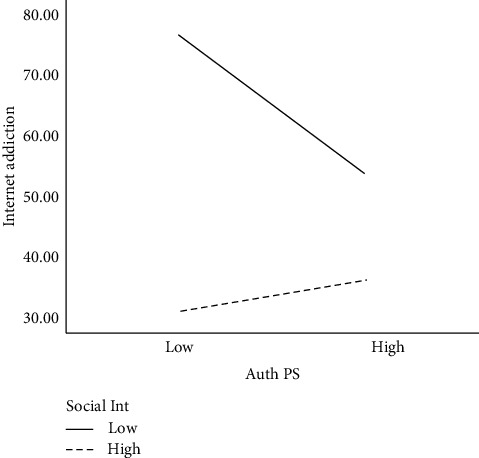
Moderating effect of social intelligence in the relationship between authoritarian parenting style and Internet addiction. *Note*. Auth PS = authoritative parenting, social int = social intelligence.

Figure 1 shows that IA is significantly higher for individuals with low SI and higher authoritarian PS than for individuals with high SI but low authoritarian PS. Figure 1 also indicates that IA is significantly lower for students with higher SI and low authoritarian PS than individuals with high SI but with high authoritarian PS.

## 4. Discussion

The study investigated the moderating role of SI in the relationship between PS and IA among undergraduate students during a lockdown due to Nigeria's global pandemic (coronavirus). The findings from the study showed that permissive parenting has a significant negative relationship with Internet addiction. This showed that students who experienced a permissive parenting style from their parents tend to exhibit a low level of Internet addiction. Although other studies found a positive relationship between permissive PS and IA [30], others [31] found that permissive and authoritative PS had a minimal impact on IA. The contradictory findings could reflect differences in cultural rearing practices between the West and African culture.

First, parental child-rearing styles carry a different meaning for different cultures and, indeed, for different families. Second, the high level of imposition among Nigerian parents might be giving way to the forces of Westernization [32, 33]. This is possible as more Western ideas such as the Internet are becoming unavoidable daily. Researchers [34] noted that while Chinese parents score very high on authoritarian parenting scales, they may express behavioural patterns that reflect a different set of norms, values, and beliefs compared with Western ideas of authoritarianism described in Baumrind's typology. The concept of control-freedom is sensitive to cultures and varies in different cultures. Permissive PS is mainly practised in the Western world than in African culture.

The introduction of information technology in the Nigerian educational system, healthcare, transportation, and many aspects of their lives, including the lockdown imposed on the nation, leaves many young people with little to do other than getting engaged with the Internet. Moreover, significant redundancy increases the time spent on the Internet for tech-savvy youths who are the primary consumers of modern technologies [35]. In addition, it could be seen that when Nigerian parents trust that their children are responsible and usually make nondetrimental decisions, they employ a permissive parenting style. Therefore, little monitoring or scrutiny of children's activities could decrease the sense of habitual curiosity and frequent Internet usage, consequently, low Internet addiction. Moreover, permissive parenting could allow children to engage in diverse activities, broadening their engagement choices, therefore paying less attention to Internet activities.

The global narrative reflects similar patterns. ICTs, while acting as catalysts for innovation, have also become subjects of scrutiny due to their potential psychological repercussions. Wickramasinghe [36] underscored this duality by revealing that while specific ICT dimensions, such as social media and Internet usage, can bolster psychological well-being, others, such as video gaming, might exert negative influences [37]. This intricate relationship between ICTs and psychological health is further elaborated by the digital life balance (DLB) scale, introduced by Duradoni et al. [38]. The DLB scale underscores the significance of balancing ICT usage's positive (harmonic) and negative (disharmonic) aspects. Maintaining equilibrium between our online and offline existences is imperative for overall well-being.

Furthermore, the result of the study did not find a significant relationship between authoritarian PS and IA. This is inconsistent with the previous research [14, 15, 31], which confirmed that authoritarian PS had the most effect on problematic Internet use. The reason for the difference could be the mean age of participants. This research was conducted on youth with a mean age of 20.65 years, who were less controllable and would want to defile orders by any means possible than those primary school children with a mean age of 9.50 in the study by Valcke et al. [39]. As such, authoritarian parenting may not be significant to them.

More so, the result of the study found a significant negative relationship between SI and IA. Our study is among the few empirical studies that have tried to link social intelligence and IA to the best of our knowledge. The inverse relationship between social intelligence and IA is understandable because young adults, who perform well in a social context, fulfil their social needs in the real world. As such, they are not motivated to use the Internet to escape from real-life social interaction or to fulfil an unmet social need. Addiction to the Internet becomes more potent when it has a functional value for the individual. On the contrary, the less socially intelligent an individual is, the more likely he is to get addicted because the Internet serves a functional purpose for him/her and helps him or her overcome real-life interpersonal difficulties.

Finally, the study aimed to determine whether SI moderated the parenting style-IA relationship during the global pandemic that forced students to be at home with their parents. We found that SI significantly moderated the relationship between only authoritarian PS and IA. The graph (see Figure 1) shows that students who are low in SI but who experienced higher authoritarian PS are more likely to be addicted to the Internet compared to students with high SI and lower authoritarian PS. This could result from their poor social skills and the authoritarian PS they experience that do not know whom to engage with them. This gives them little options than to engage more in the supposed nonsupervised virtual world where they can surf on their interests and pleasures. Thus, the Internet acts as a lubricant to cope with missing or unfulfilled social needs in one's life, and the continuous use of the Internet may lead to Internet addiction. Since the global pandemic, the lockdowns and transition of many daily activities to online have not made it easier for those with low social intelligence, as most of them now spend more time using the Internet and further create a gap in the chance of people working on their social skills and human interaction. On the other hand, the second slope (see Figure 1) revealed that students who are high in SI but have experienced low authoritarian parenting are less likely to be addicted to the Internet compared to students with high social intelligence and high authoritarian parenting. This indicates that even with high social intelligence, a greater level of authoritarian parenting will result in a higher level of IA. Besides low authoritarian parenting, the tendency to be addicted to the Internet will be minimal.

### 4.1. Implications

The findings of the present study have reasonable implications for practice. The current global pandemic has exposed some weaknesses in the child-parent relationship. Parents and children increasingly grow apart and have little to do or engage in daily interactions. The results also suggest that certain freedom and trust given to young adults could allow them to engage more in fundamental human interactions in their environment than harsh and unaffectionate PS, which could have a reverse effect such as IA. This could be useful for psychologists in helping parents improve, adjust, and eventually give up improper rearing styles. In this way, their children will grow up in a good psychosocial environment and develop sound self-esteem, thus reducing the incidence of IA. In addition, SI could act as a buffer in the face of maladjusted PS and rearing practices. Introducing civil education and standard daily etiquette can encourage mindfulness among young people in their interaction with others around them. Given the deep-seated communal values in Nigerian society, initiatives that bolster real-world human interactions and foster communal bonds can be instrumental in countering overreliance on digital interactions. Cultural programs, community gatherings, and traditional festivities, integral parts of Nigerian culture, can serve as platforms to promote these values.

### 4.2. Limitations and Future Research

One of the significant limitations of this study is the use of a cross-sectional design. Although inferences can be made about associations between dependent and independent variables, causal inferences cannot be drawn. A longitudinal design that could make for better causal inferences may need to be used for future studies.

Another potential limitation pertains to the instrument used to gauge Internet addiction. We employed the Internet addiction test (IAT) developed by Young [28], which was conceived over two decades ago despite its enduring popularity and widespread acceptance in academic circles. Since its development, the landscape of the Internet has undergone profound transformations. The rise of smartphones, social media platforms, online streaming services, and other digital innovations have reshaped how individuals interact with the online realm. While the IAT offers a robust framework to understand general Internet addiction patterns, its age might render it less attuned to capturing the nuances of contemporary Internet behaviours and dependencies. The continued use of the IAT in recent literature supports its relevance and applicability, suggesting that its core principles remain pertinent despite the dynamic digital landscape.

Second, the participants were university students. In replicating this study, other student populations should be targeted to generate a more solid relationship among constructs examined in this study because the generalisation of the results is limited to only undergraduate students.

Beyond the immediate scope of our study, the intricate nexus between IA, PS, and SI can potentially be influenced by broader digital life dynamics. The recently conceptualised digital life balance (DLB) scale by Duradoni et al. [38] offers an innovative lens to examine the harmonic and disharmonic facets of ICT usage. Furthermore, delving into the distinct impacts of various ICT platforms, as highlighted by Wickramasinghe [36], might enrich our comprehension of the IA phenomenon. Another avenue worth exploring is the relationship between IA and Internet gaming disorder.

## 5. Conclusion

In conclusion, our study has revealed several insightful associations between parenting styles, social intelligence, and Internet addiction among Nigerian undergraduates. We found that social intelligence is a vital moderator between parenting styles and Internet addiction, underscoring the importance of fostering more vital social skills among youths to mitigate Internet addiction. Notably, while the study was conducted during the pandemic, transitioning towards normalcy following a global pandemic, the findings offer unique perspectives on Internet addiction in a world forever altered by the experience. The pandemic, undoubtedly, has transformed our relationships with digital media, and its effects might have lingering influences on our behaviours and habits. This transition, which has seen an upsurge in the use of Internet services for educational and social purposes, might have implications for Internet addiction. This research re-emphasises the importance of a balanced and nuanced understanding of parenting styles and their effects on IA, especially in challenging circumstances such as a pandemic. The study also highlights the potential of SI as a protective factor, suggesting further avenues for preventive strategies against IA. As the boundaries of our digital and physical worlds continue to blur, especially for young adults, these findings provide valuable insights for educators, parents, and policymakers striving to minimise the potential downsides of digital life.

## Figures and Tables

**Table 1:** Means, standard deviations, and correlations among the study variables.

	Variables	M	SD	1	2	3	4	5	6	7	8
1	Gender			—							
2	Age	21.61	3.37	−0.06	—						
3	Who you live with			−0.07	0.16^*∗*^	—					
4	Authoritative PS	17.71	4.17	0.10^*∗*^	−0.03	−0.06	—				
5	Permissive PS	17.84	3.22	−22^*∗∗*^	0.08	0.01	0.23^*∗∗*^	—			
6	Authoritarian PS	18.41	3.12	0.03	−0.24^*∗∗*^	−0.05	0.16^*∗∗*^	0.03	—		
7	Social intelligence	93.62	13.29	−0.06	−0.01	0.03	0.11^*∗*^	0.25^*∗∗*^	0.09	—	
8	Internet addiction	68.53	19.21	−0.05	−0.01	0.05	−0.12	−0.32^*∗∗*^	−0.05	−0.42^*∗∗*^	—

*Note.* ^*∗*^= *p*  <  0.05 (two-tailed), ^*∗∗*^= *p*  <  0.01 (two-tailed), authoritative PS = authoritative parenting style, permissive PS = permissive parenting style, and authoritarian PS = authoritarian parenting style; gender was coded 0 = male and 1 = female; who you live with: 0 = parents and 1 = other guardians.

**Table 2:** Moderated multiple regression results for the moderation of social intelligence on the relationship between parenting style and Internet addiction.

Variables	Step 1	Step 2	Step 3	Step 4
*Controls*				
Gender	−0.05	−0.06	−0.09	−0.12
Age	0.01	0.02	0.03	0.03
Whom you live with	−0.05	0.05	0.06	0.08

*Main effects*				
Authoritative PS		−0.04	−0.04	−0.03
Permissive PS		−0.24^*∗∗*^	−0.24^*∗∗*^	−0.25^*∗∗*^
Authoritarian PS		−0.03	−0.04	0.04
Social intelligence			−0.32^*∗∗∗*^	−0.33^*∗∗∗*^

*Interaction effects*				
Authoritative PS X SI				0.06
Permissive PS X SI				−0.08
Authoritarian PS X SI				0.25^*∗∗*^
∆*R*^2^	0.003	0.04	0.07	0.09
∆*F*	0.78	4.41^*∗∗∗*^	18.78^*∗∗∗*^	5.11^*∗∗∗*^

*Note.* ^*∗∗∗*^= *p*  <  0.001 (two-tailed), ^*∗∗*^= *p*  <  0.01 (two-tailed); authoritative PS = authoritative parenting style, permissive PS = permissive parenting style, and authoritarian PS = authoritarian parenting style; gender was coded 0 = male, 1 = female; who you live with: 0 = father and mother, 1 = father alone, 2 = mother alone, and 3 = guardian ^*∗∗∗*^= *p*  <  0.001.

## Data Availability

The data that support the findings of the study are available from the corresponding author upon reasonable request.
